# The Clinical Laboratory: Its Value in the Actual Treatment of the Patient

**Published:** 1912-11-23

**Authors:** 


### THE CLINICAL LABORATORY.

### Its Value in the Actual Treatment of the Patient.

The clinical laboratory is now an essential part of the equipment of every modern hospital. In almost every branch of medicine laboratory aids to diagnosis are indispensable. In many of the larger teaching hospitals the laboratory work is delegated to a special official, but in most hospitals there is no such expert at hand and the work must be done by the physicians and residents. This series of articles is intended to assist the clinician by showing where the laboratory can help him to treat his patients. Very useful routine chemical and histological work can be done by any industrious man who has passed through a conscientious medical course. To be able to do most of the really useful laboratory work a man does not require to be an expert bacteriologist skilled in the cultivation of delicate micro-organisms, but he must be absolutely *au fait* with the various facts which spell contamination. He does not require to be a chemist, but he must understand the delightful simplicity of the normal solutions employed in volumetric analysis. He does not require to be deeply read in immunity and in chemical theory, but he must have at hand a few reliable works of reference. There are numerous books which have been published as a guide to laboratory technique. Many are too small, and do not warn one sufficiently of the ever-present liability to error in technique or the limitations of the methods employed. Emerson's "Clinical Diagnosis" is one of the very best. It is almost complete, as it gives chemical, microscopical, and bacteriological technique in sufficient detail. Muir and Ritchie's "Bacteriology," Sutton's "Volumetric Analysis," and Mallory and Wright's "Pathological Technique" are all worth a place in the laboratory.

The first thing which must be ascertained is the accuracy of one's methods. Before diagnosing malaria in a patient from the tropics with remittent fever, examine a stained film of one's own blood and notice how like a malaria parasite a blood platelet can look when it is deposited on the top of a red corpuscle. Before charting blood counts of a patient, count healthy blood till constant results are obtained. Before relying upon the undoubted existence of *B. coli* in a catheter specimen obtained by the sister from a female patient, examine one or two specimens obtained by the same sister from patients who are not suspected of colon bacillaria. Before attempting volumetric analysis on clinical material be sure that the standard solutions are correct. In fact, always do "controls." In the old-fashioned clinical work the only control was the dead-house or the operating theatre. The great value of the laboratory is that one can perform experiments and check results as they are obtained. The present series of articles is designed as an indication of the laboratory methods which are of direct value in treatment.

### Diseases due to Animal Parasites.

The only diseases due to animal parasites which are of importance in this country are dysentery, malaria, the various diseases due to worms, and syphilis.

*Dysentery.*—Cases of dysentery from the tropics are fairly common, even in the inland towns of England. Both the bacillary and the amœbic forms may be encountered. It is of importance to differentiate between the two, for there is now no doubt that the ipecacuanha treatment is to a certain degree specific for the amœbic form. Indeed, Rogers has recently brought forward very good evidence that emetine itself is the specific element in the treatment. The amœbæ must be found in the stools to establish the diagnosis.

A certain degree of familiarity with the normal constituents of the fæces, as well as with the use of the microscope on unstained material, is desirable. The stool must be examined whilst still warm. Some of the mucus should be examined and large cells searched for. A typical example of the entamœba histolytica should, before it is accepted by one who is not an expert, show (1) engulfed red corpuscles; (2) amœbic movement. Such a cell having been found we know that we are dealing with amœbic dysentery, and in addition to being put on our guard against liver abscess, we have good reason for thinking that the ipecacuanha or emetine treatment will do good.

*Malaria.*—The subject of malaria is one which exemplifies the danger of a little knowledge. As Manson points out so emphatically, every intermittent fever from the tropics is not malaria, yet it is very easy for a novice to find in a blood film quite passable substitutes for the parasites. A very good stain—in the writer's opinion the best for routine blood work of all kinds—is Giemsa's stain, made up as follows: One of Burroughs Wellcome's Eosin Azur tabloids is dissolved in $2\frac{1}{2}$ c.c. of glycerine and $7\frac{1}{2}$ c.c. of pure methyl alcohol (Merck's). The blood film is fixed in methyl alcohol for about one minute, then placed in a staining trough and 8 to 10 drops of the stain placed on the film. This is left for one minute, and then 5 or 6 c.c. of distilled water are added and the whole mixed by gently shaking the trough. After two or three minutes the film is rapidly washed in distilled water and dried between filter-paper. The specimens of the stain vary slightly, and so do, apparently, the films, and it is impossible to state the times with absolute accuracy. A trial can always be made with a spare film. If the blood platelets show a definite ruby-red centre and a pale-blue halo the film is correctly stained.

*Diseases due to Worms.*—In this country the diagnosis is usually made by the patient, but there are cases of obscure anæmia, especially in miners and cases from the tropics, where it is desirable to exclude ankylostomum. It is not sufficient merely to place a small fragment of fæces under a cover slip and examine. The stool should be emulsified in water and centrifugalised, this operation being repeated several times. The sediment is then examined for ova.

A minor point is, in looking for the eggs of *ascaris*, to avoid mistaking for ova the soap masses which are so often found in the stools. The resemblance is very striking.

*Syphilis.*—Laboratory work has revolutionised syphilology. The diagnosis by means of the examination for the parasite in the primary stage, the Wassermann reaction in the later stages, and in obscure cases for its diagnostic value, and at all stages as a guide to the efficacy of treatment, are a host in themselves, without claiming salvarsan as a product of laboratory work.

*Demonstration of the Spirochæte.*—The primary sore is usually the lesion which has to be examined for the spirochæte. An excellent way is to take a piece of the excised chancre and make smears from the cross section of the ulcer on a slide. Fix in absolute alcohol for half an hour (or methyl alcohol for a minute) and then stain with Giemsa as indicated above, except that instead of adding distilled water use a 1: 1,000 solution of sodium carbonate and leave this for fifteen minutes. The nuclei of the leucocytes should be stained a black red. If these are lightly stained it is waste of time and eyesight to look for the spirochæte. Other methods of demonstrating the spirochæte are (1) the Indian-ink method of Burri, (2) the dark-ground condensor. Both of these are specialists' methods. The value of an examination for spirochætes is enormous. It means that treatment can be commenced before the body is flooded with infecting organisms.

It is now almost criminal to wait for secondary symptoms before commencing treatment, likewise —with the possibility of making an accurate diagnosis—it is not right to put a patient on immediate treatment with mercury merely because he has a suspicious sore.

The Wassermann reaction is not a suitable subject for a small laboratory or a pathologist without experience of serum work. If anyone is desirous of trying his hand at the test he will find full directions in Emerson's book. This account gives all the necessary controls. A careful perusal of this article should persuade the cautious clinician that he must not accept a report unless it comes from an absolutely reliable worker. There are numerous short cuts which have been published by different workers. All of them have an increased liability to error, and it is noteworthy that most of the errors lie in the direction of giving a positive reaction where there should be a negative.

Syphilitic liver extract is now very difficult to obtain, but the reaction is almost as delicate if Sach's " Antigen " is used, and this can be obtained commercially. The hæmolytic serum required can also be obtained, but it is always necessary to " titrate " it. The novice should commence on a series of sera, including one absolutely free from suspicion of syphilis, and one or two from florid syphilis in the secondary stage. What is the value of the reaction? All points are not yet settled, but it may be stated:

1. In the primary stage it is usually negative.
2. In the secondary stage it is practically always positive.
3. If the case has been treated till all symptoms have vanished but the reaction remains positive, further treatment is required.
4. If all symptoms have vanished and the reaction is negative, the patient may be given a rest, but the reaction should be watched.
5. If there is any urgent reason to discover whether recovery is perfect in an old syphilitic (question of marriage) the reaction should be performed. If negative a small injection of salvarsan should be given. If cure is not attained this will probably cause a transient return of the reaction.
6. Tertiary lesions may or may not give a reaction.
7. Cases of early tabes or general paralysis practically always give a reaction. In many cases a negative reaction is desirable before a diagnosis of neurasthenia is made in a middle-aged man.

